# Late‐Onset Invasive Aspergillosis With Pituitary Involvement and Dysfunction Following CD19 Chimeric Antigen Receptor T‐Cell Therapy

**DOI:** 10.1002/jha2.70138

**Published:** 2025-09-02

**Authors:** Daisuke Ikeda, Tomohiro Nawada, Takumi Kondo, Takayuki Shinohara, Tomohiro Nagano, Saya Kubota, Ryuichiro Hiyama, Masaya Ueno, Hiroki Kobayashi, Keisuke Seike, Hideaki Fujiwara, Noboru Asada, Daisuke Ennishi, Keiko Fujii, Nobuharu Fujii, Masanori Makita, Yoshinobu Maeda

**Affiliations:** ^1^ Department of Hematology, Oncology and Respiratory Medicine Okayama University Graduate School of Medicine, Dentistry and Pharmaceutical Sciences Okayama Japan; ^2^ Department of Hematology and Oncology Okayama University Hospital Okayama Japan; ^3^ The Center for Graduate Medical Education Okayama University Hospital Okayama Japan; ^4^ Department of Fungal Infection National Institute of Infectious Diseases Tokyo Japan; ^5^ Department of Hematology Okayama City Hospital Okayama Japan; ^6^ Department of Hematology and Oncology Japanese Red Cross Society Himeji Hospital Hyogo Japan; ^7^ Center For Comprehensive Genomic Medicine Okayama University Hospital Okayama Japan; ^8^ Division of Clinical Laboratory Okayama University Hospital Okayama Japan; ^9^ Division of Transfusion and Cell therapy Okayama University Hospital Okayama Japan; ^10^ Department of Hematology Chugoku Central Hospital Hiroshima Japan

**Keywords:** aspergillosis, CD19 CAR T, invasive fungal infection, pituitary

## Abstract

**Introduction:**

Invasive fungal infection (IFI) after chimeric antigen receptor (CAR) T‐cell therapy is less common than bacterial and viral infections, but can be fatal once it develops. As most cases occur within 30 days after CAR T‐cell infusion, late‐onset IFI—particularly mould infection—appears to be under‐recognised.

**Discussion:**

We report an illustrative case of pituitary aspergillosis developing as late as one year after CD19 CAR T‐cell therapy, highlighting a persistent risk in certain patients with delayed immune reconstitution.

**Conclusion:**

This case underscores the need for continued vigilance and individualised antifungal strategies to prevent IFI beyond the early post‐infusion period.

**Trial Registration:**

The authors have confirmed clinical trial registration is not needed for this submission.

## 1 Introduction

A growing body of evidence suggests that infection is the leading driver of non‐relapse mortality after chimeric antigen receptor T (CAR T)‐cell therapy [1]. The burden of CAR T‐cell‐associated infections is largely phase‐specific: The acute phase (0–28 days), delayed phase (29–90 days) and the late phase (>90 days), which are defined based on the time after infusion [2]. The underlying immune deficits vary between different periods, with the main contributors to infection shifting from neutropenia early to hypogammaglobulinemia and protracted CD4^+^ T‐cell recovery later [2].

A few recent studies have consistently reported a predominance of invasive fungal infections (IFIs) in the early phase and a low frequency (0.6%–2.8%) of IFIs following CD19 CAR T‐cell therapy [3, 4, 5]. Along with the global trend against universal anti‐mould prophylaxis [6], late‐onset mould infections seem to be neglected. Here, we present an illustrative case of pituitary aspergillosis that developed as late as 1 year after CD19 CAR T‐cell therapy, along with longitudinal monitoring of immune reconstitution.

## 2 Case Description

A 51‐year‐old man with a history of dyslipidaemia was diagnosed with advanced‐stage diffuse large B‐cell lymphoma having a rearrangement of *IGH‐BCL2* identified by fluorescence in situ hybridisation 3 years ago. Due to the aggressive disease nature, intensive induction therapy using hyper fractionated cyclophosphamide, vincristine, doxorubicin and dexamethasone plus rituximab regimen was chosen, which effectively yielded complete remission (CR); however, the patient experienced early relapse only 4 months later. Although salvage chemotherapy followed by autologous stem cell transplantation (ASCT) re‐induced CR, the response was short‐lived, as with frontline treatment, and the patient eventually relapsed again 8 months post‐ASCT. The biopsied lymph node showed histological evidence of follicular lymphoma, with limited therapeutic options, which led us to perform three cycles of bendamustine plus obinutuzumab as bridging therapy. Despite the risk of CAR T‐cell manufacturing failure associated with recent exposure to bendamustine [7], the apheresis process was successful, with a reduction in tumour burden consistent with the near‐CR level. Based on baseline hyperferritinaemia and neutropenia, the CAR‐HEMATOTOX score was 3, which is considered a high‐risk factor for CAR T cell‐mediated haematotoxicity [8]. Screening magnetic resonance imaging (MRI) and computed tomography did not identify any suspicious foci of IFI in the lungs or sinuses.

Following standard lymphodepletion therapy with fludarabine plus cyclophosphamide, the patient received CD19 CAR T‐cell therapy with axicabtagene ciloleucel (axi‐cel). Fluconazole (FCZ, 200 mg daily) was initiated as antifungal prophylaxis. The clinical course after CAR T‐cell infusion was uncomplicated, except for grade 2 cytokine release syndrome (CRS), which was managed with tocilizumab, allowing the patient to be discharged on day 21.

**FIGURE 1:**
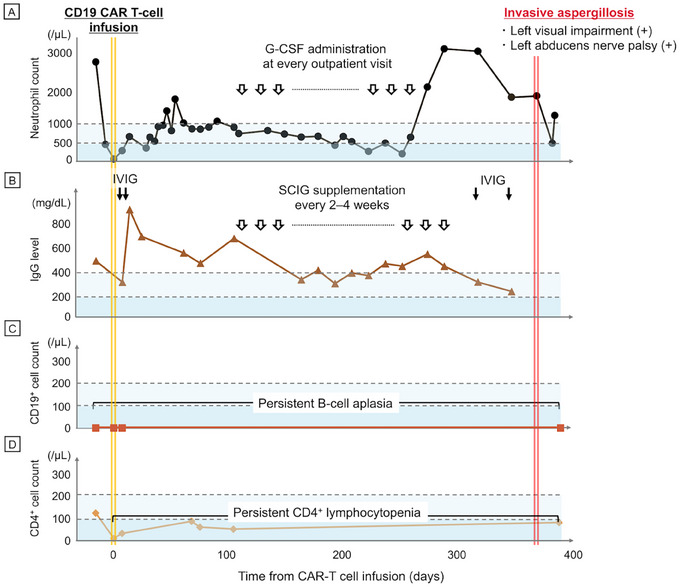
Trajectory of the patient immune state before and after CAR T‐cell infusion. (A) Absolute neutrophil counts, (B) IgG levels, (C) absolute CD19^+^ cell counts and (D) absolute CD4^+^ cell counts in peripheral blood. Pale and deep bluish areas corresponded to moderate and severe declines in each parameter, respectively. CAR, chimeric antigen receptor; G‐CSF, granulocyte‐colony stimulating factor; IVIG, intravenous immunoglobulin; SCIV, subcutaneous immunoglobulin.

However, immune reconstitution was delayed, as illustrated in Figure 1. While a biphasic pattern of neutropenia was expected, the duration of the second dip in neutrophil count, which required granulocyte colony‐stimulating factor support, extended beyond 6 months (Figure 1A). Bone marrow examination revealed no evidence of secondary haematologic malignancy. Regarding B‐cell recovery, the patient continued to require regular immunoglobulin supplementation with persistent B‐cell aplasia for more than a year (Figures 1B,C). Although CD4^+^ T cells had already been ablated by prior treatments, including bendamustine, CD4^+^ lymphocytopenia became more profound, dropping to less than 100/µL (Figure 1D). FCZ was consistently prescribed at the time of lymphodepletion therapy for one year, but was not switched to a mould‐active agent. Serum galactomannan levels were not routinely measured.

**FIGURE 2:**
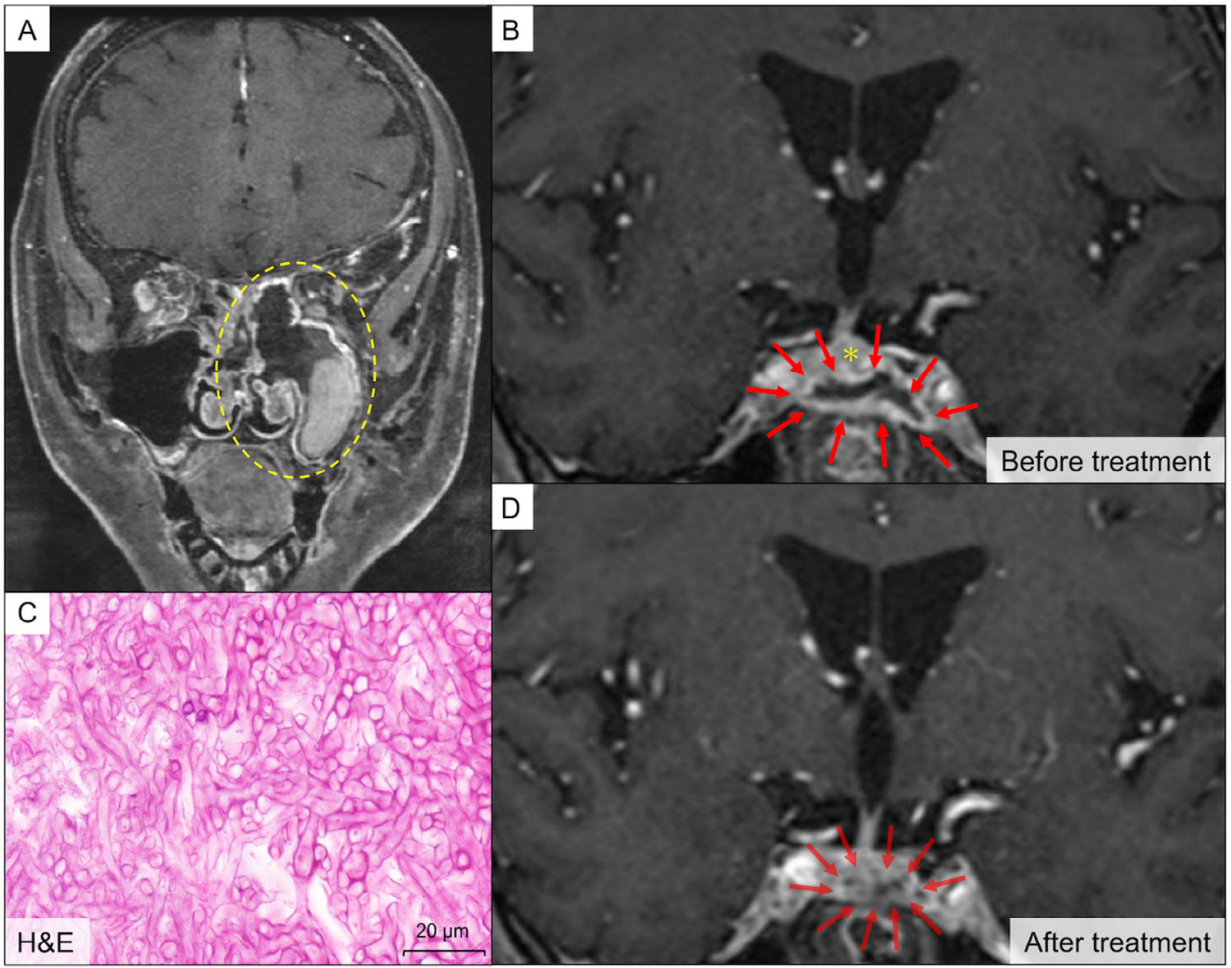
Radiological and pathological findings of invasive aspergillosis. (A) and (B) T2‐weighed MRI with contrast enhancement at the onset of infection, showing views of the paranasal cavity and cavernous sinus, respectively. The wall of left submandibular and ethmoid sinus was destroyed by the fungal ball (yellow dotted circle). Red arrows indicate the border of the abscess with contrast enhancement. A yellow asterisk marks the enlarged pituitary gland. (C) Histological findings of the resected fungal ball. (D) Contrast‐enhanced MRI 3 months after antifungal treatment. Light red arrows indicate the original abscess wall, whose margins have become indistinct. H&E, haematoxylin and eosin staining; MRI, magnetic resonance imaging.

Approximately 1 year after axi‐cel infusion, during which CR was sustained, the patient experienced preceding sinus headaches and was subsequently admitted to a community hospital because of left visual impairment and left abducens nerve palsy. MRI revealed a bulky mass occupying the left maxillary and sphenoid sinuses, which appeared T2 hypo‐to‐iso intense, mixed with haemorrhage‐associated hyperintensity, and was accompanied by bony destruction of the nasal cavity (Figure 2A). Contrast‐enhanced MRI revealed that the infection extended to the cavernous sinus with epidural abscess formation, leading to pituitary enlargement (Figure 2B). Endoscopic sphenoidectomy was performed to debulk the suspected fungus ball and confirm the diagnosis. The resected specimen showed numerous septate mould hyphae (Figure 2C), positive for Grocott staining. While fungal culture was not carried out, DNA was extracted from formalin‐fixed paraffin‐embedded samples using the QIAamp DNA FFPE Tissue Kit (Qiagen, Hilden, Germany), and polymerase chain reaction (PCR)—performed using a T100 Thermal Cycler (Bio‐Rad, Hercules, CA) and targeting the internal transcribed spacer (ITS), D1/D2 and β‐tubulin gene regions [9]—identified a sequence with high homology to *Aspergillus fumigatus*. Unfortunately, surgical drainage of the abscess was deferred because of the high risk of fatal bleeding from the left internal carotid artery. After voriconazole (VRCZ) was initiated with a high target trough concentration of > 2 µg/mL for central nervous system (CNS) infection [10], the patient was transferred to our centre.

**FIGURE 3:**
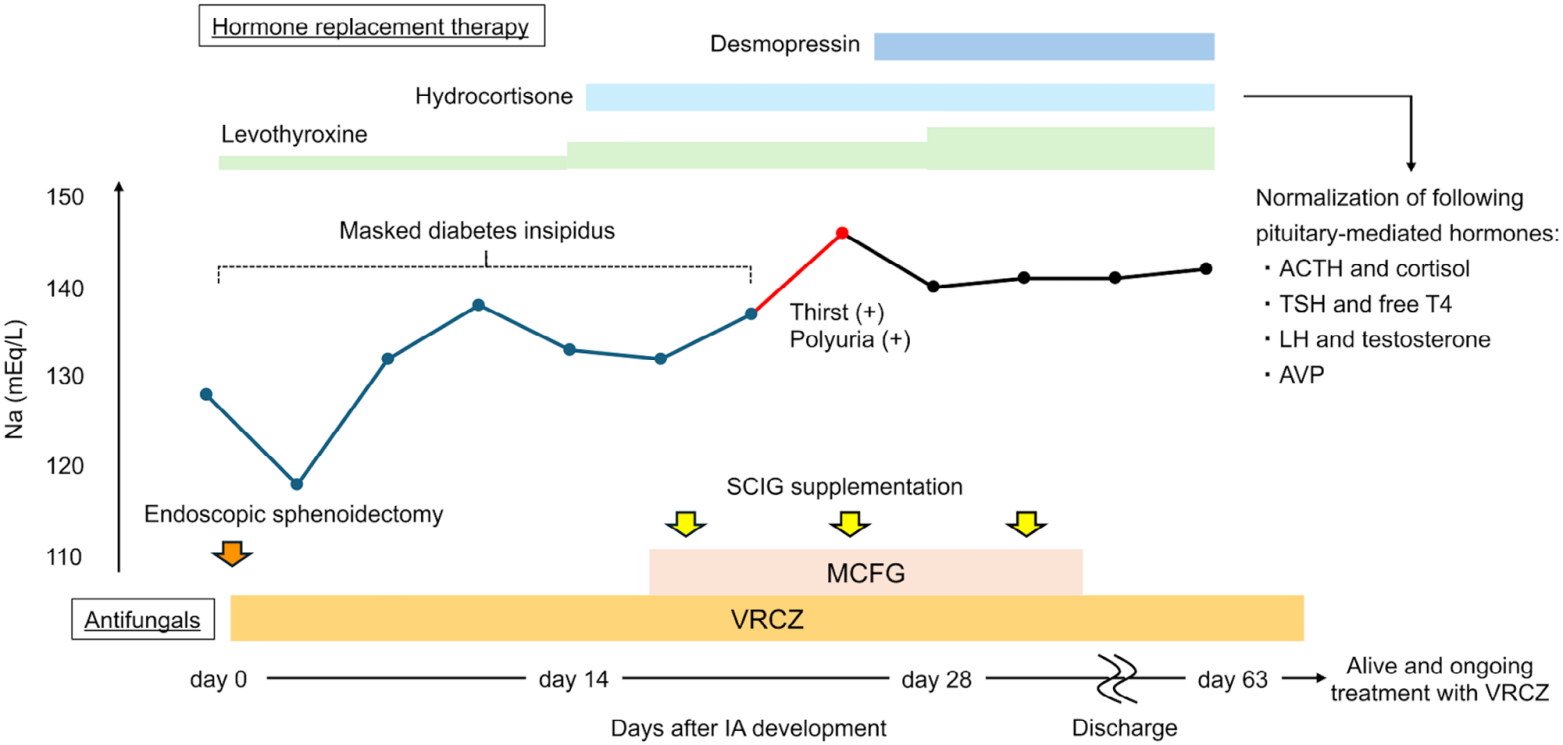
Clinical course and longitudinal serum sodium dynamics during treatment for pituitary aspergillosis. Blue, red, and black lines correspond to lower, higher, and stabilised serum sodium levels, respectively. Hydrocortisone improved adrenal insufficiency‐associated hyponatremia, which in turn unmasked diabetes insipidus‐related hypernatremia. Abbreviations: ACTH, adrenocorticotropic hormone; AVP, vasopressin; IA, invasive aspergillosis; LH, luteinizing hormone; TSH, thyroid‐stimulating hormone; T4, free thyroxine 4; MCFG, micafungin; Na, sodium; SCIG, subcutaneous immunoglobulin; VRCZ; voriconazole.

Given the concerns regarding pituitary dysfunction, a comprehensive hormone panel was tested. Thyroid‐stimulating hormone (0.6 mIU/L) and luteinizing hormone (0.4 mIU/mL) levels were decreased, which paralleled the decline in downstream hormones, such as free thyroxine 4 (0.65 ng/dL) and testosterone (<0.09 ng/mL). Secondary adrenal insufficiency developed 14 days after hospitalisation. Despite markedly decreased morning cortisol levels (0.1 µg/dL), the pituitary‐adrenal axis feedback was impaired, with adrenocorticotropic hormone (ACTH) remaining at lower levels (7.4 pg/mL) around the normal range (7.2–63.3 pg/mL). Regarding posterior pituitary hormones, vasopressin levels decreased to undetectable levels (<0.4 pg/mL). Prompt hormone replacement therapy with levothyroxine and hydrocortisone could prevent thyroid and adrenal crises and improve the associated hyponatraemia (Figure 3). Antifungal therapy with VRCZ combined with micafungin during hospitalisation resolved cranial nerve palsy and normalised pituitary hormone levels. Follow‐up imaging performed 3 months after discharge showed marked shrinkage of the abscess within the cavernous sinus (Figure 2D). The patient is alive 10 months after the onset of IFI and continues to receive continuous VRCZ treatment.

## 3 Discussion

Our understanding of IFI risk after CAR T‐cell therapy has expanded in recent years, but remains limited to those occurring at earlier time points. A landmark study by Little et al. showed that, even without routine antifungal prophylaxis, the 100‐day cumulative incidence of IFI was only 1.8% among 280 patients with B‐cell non‐Hodgkin lymphoma (B‐NHL) who received CD19 CAR T‐cell therapy. More recently, French registry data identified 32 IFI cases among 1144 patients (2.8%) with B‐cell malignancies [5]. Notably, approximately half of the cases (*n* = 14, 43.7%) occurred in the later phases (30–100 days in 8 patients [25.0%] and >100 days after infusion in 6 patients [18.7%]). In line with this finding, a systematic review reported that among 32 mould infections in 2686 recipients of CAR T‐cells, 47% occurred 30 days after infusion [11]. These data support the idea that IFI risk may not simply diminish over time, which sheds light on the nuanced antifungal prophylaxis strategy currently proposed. Many guidelines emphasise early period‐specific factors as key IFI risks, such as profound neutropenia after lymphodepletion therapy and the dose and duration of corticosteroids for CRS, and are largely extrapolated from experience with allogeneic transplantation [6]. In the present case, the prolonged duration of grade 3–4 neutropenia following the initial haematopoietic recovery was likely to have made a substantial contribution to the development of IFI. However, the 1‐year latency after the nadir of neutropenia led us to speculate that neutropenia alone is not sufficient and that a constellation of humoral and cellular immune dysfunction may be required to develop CAR T‐associated IFI. Similar to our case, a recent study showed that 39.1% of patients with B‐NHL never displayed B‐ and T‐cell lineage immune reconstitution even 1 year after infusion and that higher CAR‐HEMATOTOX scores were likely to be categorised into this no‐recovery group [12]. Further studies with an in‐depth characterisation of immune determinants are warranted to refine risk stratification and pave the way for tailored antifungal strategies, as proposed by the Memorial Sloan Kettering Cancer Center group [3].

Another unique aspect of our case is the description of a granular clinical scenario of secondary pituitary aspergillosis after CAR T‐cell therapy for the first time. Although CNS aspergillosis is rare, direct dissemination from the sinuses to the intracranial space is anatomically plausible [13]. The mortality of CNS aspergillosis has historically been dismal, with a reported survival rate of only 31% following VRCZ treatment, and it is especially poor in patients who have not undergone neurosurgical intervention [14]. Even in the modern era, the CEREALS study showed that the 6‐week mortality of CNS aspergillosis was 45% [15]. Despite severe net immunosuppression, we successfully treated the patient with antifungal drugs alone, without neurological sequelae. It remains uncertain whether there is a difference in the susceptibility to severe disease as an underlying predisposition to allogeneic transplantation and CAR T‐cell therapy. We believe that the immediate initiation of VRCZ and prompt supplementation of deficient hormones to maintain the general condition may have contributed to the favourable outcomes. Although a single case report does not provide sufficient evidence to change general management, the rare but high fatality rate of post–CAR T fungal infections (19.4%) reported in a large meta‐analysis [1] underscores the need for enhanced accumulation of detailed clinical data.

In conclusion, although rare, a subset of patients may remain at risk of invasive aspergillosis even after 365 days of CAR T‐cell therapy. Increased awareness and careful surveillance are necessary until long‐term follow‐up data are available.

## Author Contributions

DI prepared an original draft of the manuscript. DI, TN, and TK were responsible for the overall treatment. TS performed PCR to identify the pathogen. Tomohiro Nagano, SK, RH, MU, HK, KS, HF, NA, DE and NF supported treatment during hospitalisation. KF performed apheresis of CAR T‐cell therapy. MM managed the patient in an outpatient setting after discharge. YM supervised the study.

## Ethics Statement

Ethical approval was not required because written informed consent for publication was obtained from the patient.

## Consent

Written informed consent was obtained from the patient to publish this case.

## Conflicts of Interest

DE received honoraria from Bristol Myers Squibb. NF received honoraria from Gilead Sciences and Bristol Myers Squibb. The other authors declare no conflicts of interest.

## Data Availability

The data that support the findings of this study are available from the corresponding author upon reasonable request.
